# The rise of AMPylation: from bacterial beginnings to modern implications in health and disease

**DOI:** 10.1042/BST20253056

**Published:** 2025-07-08

**Authors:** Meghomukta Mukherjee, Anju Sreelatha

**Affiliations:** 1 Department of Physiology, University of Texas Southwestern Medical Center, Dallas TX 75390, U.S.A.; 2 Charles and Jane Pak Center for Mineral Metabolism and Clinical Research, University of Texas Southwestern Medical Center, Dallas TX 75390, U.S.A.

**Keywords:** adenylylation, AMP transferase, FIC domain, post-translational modification, unfolded protein response

## Abstract

Protein AMPylation is a post-translational modification in which adenosine monophosphate (AMP) from ATP is covalently attached to a target protein via a phosphodiester bond. This reaction is catalyzed by AMPylases, a diverse group of enzymes containing adenylyltransferase, filamentation induced by cyclic AMP (FIC), or kinase domains. As a reversible modification, AMPylation is dynamically regulated by both writer enzymes (AMPylases) and eraser enzymes (deAMPylases). Since its initial discovery in bacterial nitrogen metabolism in 1967, AMPylation has been recognized as a critical regulatory mechanism in both prokaryotic and eukaryotic systems. Recent studies link AMPylation to neurological disorders, diabetes, and cancer metastasis, underscoring its physiological and pathological significance. In this review, we present an overview of the discovery of AMPylases and deAMPylases, highlighting their role in cellular signaling, stress response, and host–pathogen interactions.

Protein AMPylation, also known as adenylylation, is the covalent attachment of adenosine monophosphate (AMP) to protein substrates. Analogous to phosphorylation, AMPylation uses ATP as a donor; however, instead of transferring the γ-phosphate, AMPylases transfer the α-phosphate via a phosphodiester linkage to the hydroxyl side chain of threonine, tyrosine, or serine residues (Figure 1). AMPylation follows the classic signaling paradigm of ‘writers’ and ‘erasers’, with deAMPylases acting to remove AMP from modified proteins. This dynamic regulation of AMPylation is a key factor in modulating enzymatic activity and protein–protein interactions.

**Figure 1:**
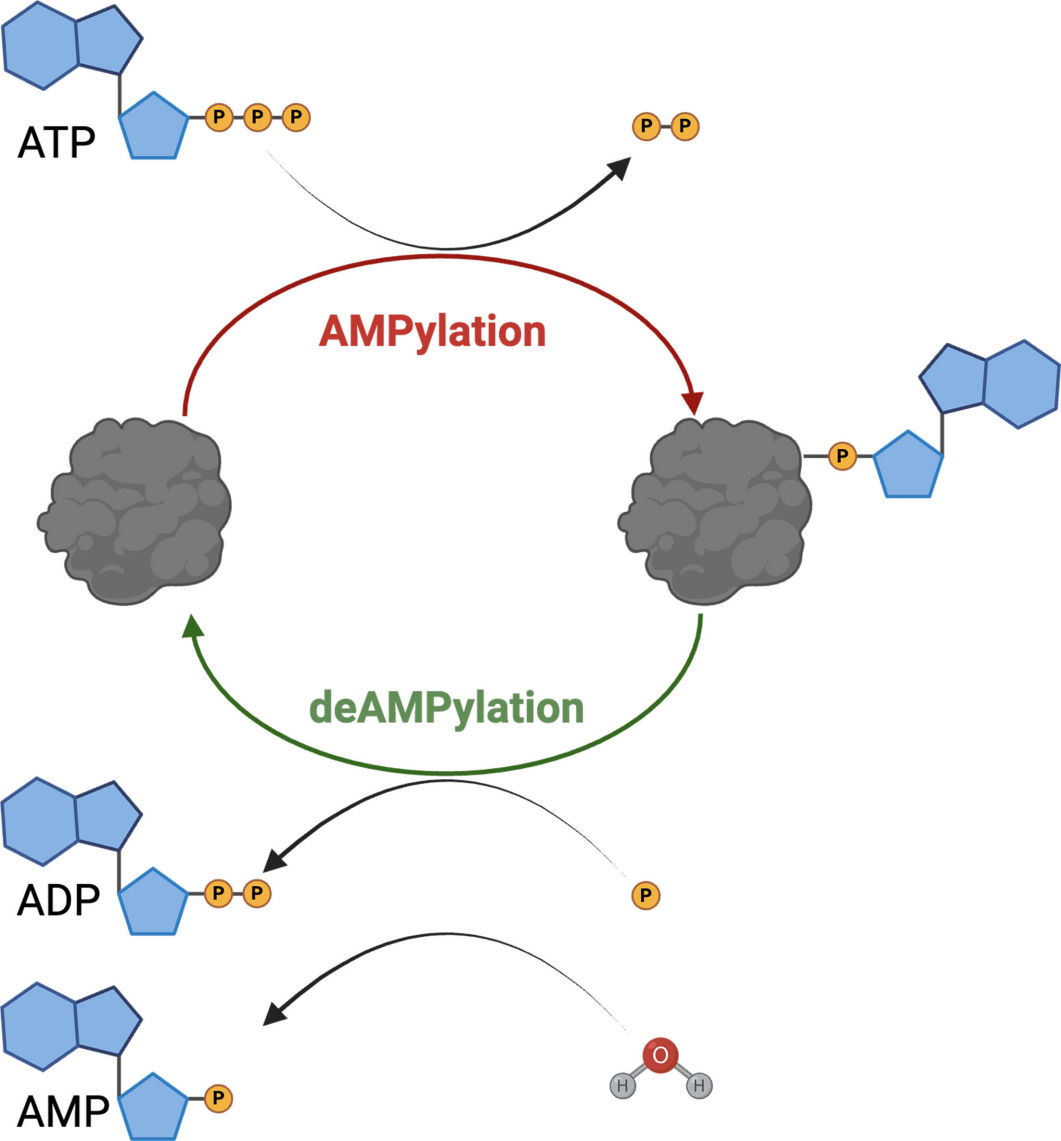
Protein AMPylation is a reversible post-translational modification. AMPylation is the covalent attachment of AMP to the hydroxyl side chain of serine, threonine, or tyrosine amino acids in protein substrates. DeAMPylation is the process of removing the AMP from protein substrates, resulting in the release of AMP or ADP through a hydrolytic or phosphorolytic reaction, respectively. Image generated with BioRender.

Since its discovery in 1967 as a post-translational modification in bacterial glutamine synthetase (GS), protein AMPylation has emerged as a significant regulatory mechanism in prokaryotes and eukaryotes [1,2]. While pathogenic bacteria exploit AMPylation to alter host cell signaling during infection, endogenous eukaryotic AMPylases play a central role in cellular metabolism and stress responses [3–5]. Recently, AMPylation has been shown to play a role in motor neuron disease, neurodevelopmental disorders, diabetes, and cancer metastasis [6–8]. Given its evolutionary conservation and expanding relevance in both physiology and disease, there is growing interest in understanding the molecular mechanisms and biological consequences of AMPylation.

In this review, we summarize the current understanding of AMPylation and its functional importance in prokaryotic and eukaryotic systems.

## Glutamine synthetase adenylyltransferase

GS catalyzes the conversion of glutamate and ammonia to glutamine. The activity of GS is tightly regulated through cumulative feedback inhibition to ensure that glutamine synthesis is finely tuned according to metabolic demands. Given its central role in nitrogen assimilation and complex regulation, *Escherichia coli* GS has been extensively studied for its kinetic and structural properties [9].

In the mid-1960s, the laboratories of Stadtman and Holzer conducted a series of biochemical studies aimed at understanding the enzymatic properties, regulation, and structural characteristics of *E. coli* GS [10]. During the purification of GS, they unexpectedly identified a distinct protein preparation that differed from the previously characterized GS [11,12]. This newly discovered form was insensitive to feedback inhibition despite identical amino acid compositions, purification methods, and assay conditions. Further investigation revealed that *E. coli* growth conditions influenced the formation of two distinct protein preparations termed GS I or GS II [13,14]. Interestingly, the absorption spectra for GS II displayed a peak at 260 nm, suggesting the presence of nucleotides [12]. Although this modification remained stable after extensive dialysis and acidic treatment, incubation with snake venom phosphodiesterase led to the release of AMP from GS II and subsequent conversion to GS I [12]. These results provided the first evidence that GS contains AMP covalently attached through a phosphodiester bond, marking the discovery of protein AMPylation (adenylylation) as a post-translational modification [11,12].

Building on this discovery, Kingdon et al. purified glutamine synthetase adenylyltransferase (GS-ATase) from *E. coli* which catalyzed the AMPylation of GS [15]. This finding aligned with earlier work by Mecke et al. who isolated a GS ‘inactivating enzyme’ from crude *E. coli* extracts [16]. Proteolytic digestion of GS incubated with GS-ATase and ^14^C-ATP revealed that the radiolabeled AMP was covalently bound to the hydroxyl side chain of tyrosine [17,18]. Furthermore, purification from *E. coli* extracts revealed that two components, GS-ATase and a regulatory protein PII, can catalyze the deAMPylation of GS in a phosphorolytic reaction generating ADP [19–24]. This reversibility highlights the dynamic nature of GS regulation in response to cellular status.

**Figure 2:**
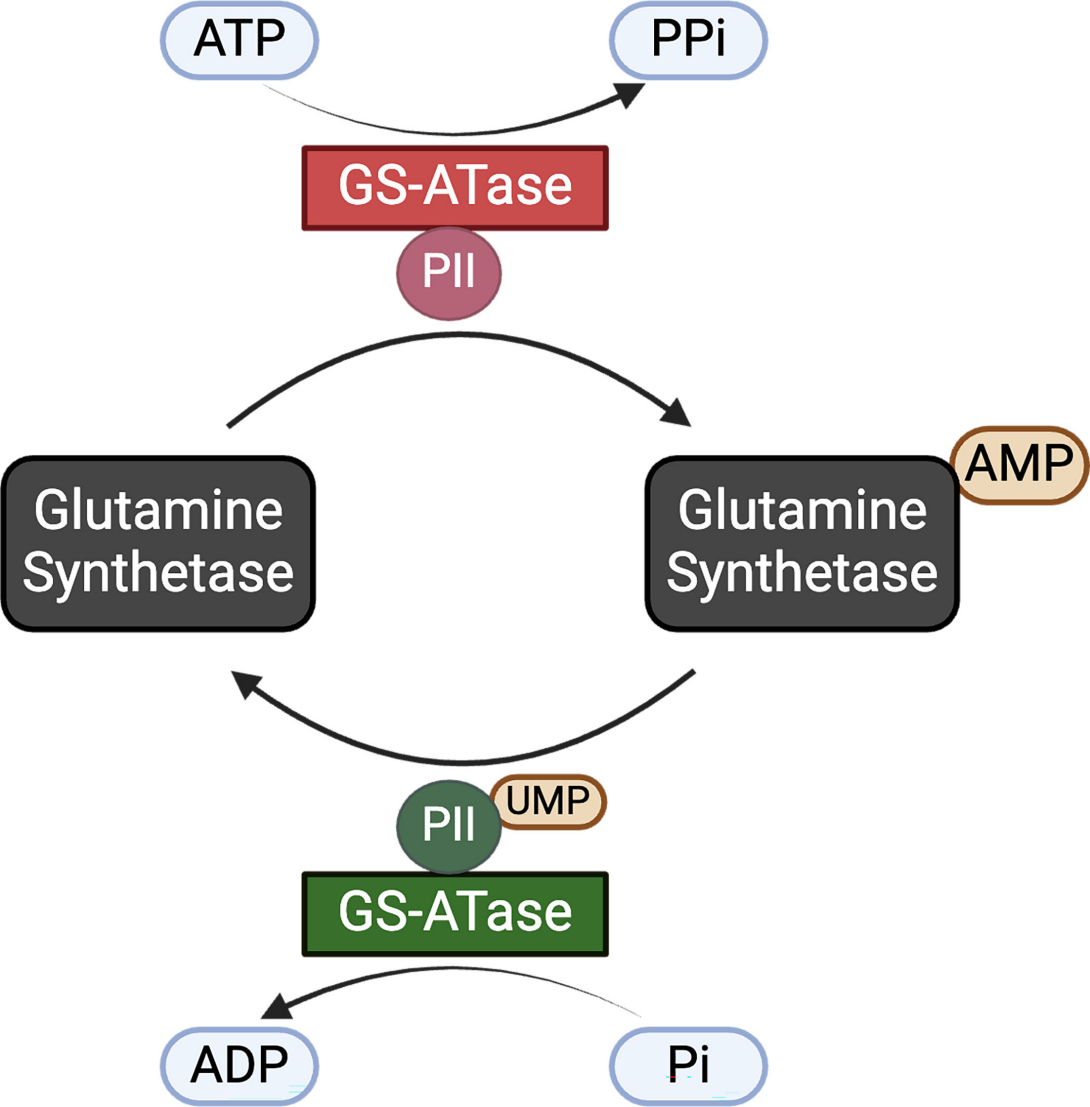
GS-ATase catalyzes reversible AMPylation of glutamine synthetase. The regulatory protein PII controls the AMPylation and deAMPylation activities of GS-ATase. The binding of PII enhances the AMPylation activity of GS-ATase, while PII-UMP promotes deAMPylation. This bicyclic cascade, consisting of adenylylation and uridylylation, is regulated by multiple metabolites of nitrogen metabolism including glutamine and α-ketoglutarate. Image generated with BioRender. ADP, adenosine diphosphate; AMP, adenosine monophosphate; ATP, adenosine triphosphate; GS-ATase, glutamine synthetase adenylyltransferase; Pi, inorganic phosphate; UMP, uridine monophosphate; PPi, inorganic pyrophosphate.

GS-ATase activity was mapped to the *glnE* gene in *E. coli*, which encodes for a ~115 kDA protein [25,26]. GS-ATase consists of two homologous domains at the N- and C-termini, linked by a central regulatory domain, each performing distinct activities [27]. The C-terminal domain catalyzes the AMPylation of GS, while the N-terminal domain facilitates its deAMPylation [28]. The switch between these two opposing activities is modulated by the regulatory protein PII, which binds to GS-ATase [29,30]. Under nitrogen starvation, PII is uridylylated by uridylyltransferase, forming PII-UMP, which interacts with GS-ATase and shifts its activity toward deAMPylation [31–33]. Conversely, when nitrogen is abundant, unmodified PII binds to GS-ATase, promoting AMPylation and reducing GS activity [31,32]. These findings represent one of the earliest identified bicyclic signal transduction systems. Through UMPylation (uridylylation) and AMPylation (adenylylation), *E. coli* dynamically adjusts GS activity based on the nitrogen availability, ensuring metabolic balance and efficient resource utilization (Figure 2) [34].

GS plays a key role in both short-term and long-term adaptation to changes in nitrogen availability [9]. Under nitrogen-rich conditions, GS-ATase AMPylates GS to reduce its activity and make it more susceptible to feedback inhibition by nitrogenous metabolites, preventing excessive accumulation of glutamine [1]. Under nitrogen-limited conditions, deAMPylation activity of GS-ATase increases GS activity, allowing the cell to efficiently assimilate nitrogen into glutamine [1]. This ensures efficient nitrogen assimilation when nitrogen is scarce and controls nitrogen over-accumulation when nitrogen is abundant.

## FIC domain

*Vibrio parahaemolyticus* is a Gram-negative bacterium responsible for gastroenteritis contracted through the consumption of raw or undercooked seafood. Pandemic strains of *V. parahaemolyticus* are particularly virulent, producing toxins and bacterial effector proteins that are injected directly into the host cell to promote bacterial survival and pathogenesis [35]. One such effector, VopS, disrupts the host actin cytoskeleton and inhibits Rho family GTPases [36,37]. Yarborough et al. conducted a pivotal experiment to investigate the molecular mechanisms by which VopS inhibits Rho family GTPases [38]. Mass spectrometry analysis of Rac GTPase co-expressed with VopS revealed an unexpected mass shift of 329 daltons, corresponding to the covalent addition of AMP to Rac [38]. This discovery established AMPylation as a novel post-translational modification used by bacterial pathogens to manipulate host cell signaling, revitalizing the field of AMPylation 42 years after the identification of GS-ATase-mediated AMPylation.

Sequence analysis of VopS revealed a C-terminal FIC domain with a highly conserved histidine residue essential for its AMPylation activity [38]. Shortly thereafter, the *Histophilus somni* protein, IbpA was also shown to harbor an FIC domain and AMPylate Rho GTPases leading to the collapse of the host actin cytoskeleton [39]. Structural and kinetic studies of IbpA and VopS revealed that the AMPylation activity requires an invariant histidine, which acts as a general base to deprotonate the hydroxyl group of either tyrosine or threonine within the switch I loop of Rho GTPases [40,41]. The catalytic loop of the Fic domain positions the α-phosphate of ATP in an optimal orientation for a nucleophilic attack by the deprotonated hydroxyl group, leading to the covalent attachment of an AMP moiety [41]. This modification prevents Rho GTPases from binding to their downstream effectors such as PAK, thereby disrupting signaling pathways required for cytoskeleton regulation [38,39].

More than 2700 FIC domain-containing proteins have been identified across prokaryotes, all sharing a highly conserved HPFx(D/E)GN(G/K)R motif [41]. However, these proteins also contain a variety of accessory domains, which likely contribute to their substrate specificity and functional diversity [42,43]. The structural variations among FIC domain-containing proteins may explain their ability to target different residues and employ distinct nucleotide donors, broadening their role in cellular regulation and host–pathogen interactions [2,4]. In contrast with prokaryotes, eukaryotes only have one FIC domain-containing protein, which is present in metazoans but absent in plants and fungi [44].

The human genome encodes for a single FIC domain-containing protein FICD, also known as HYPE (Huntingtin yeast interacting protein E) [39]. FICD contains a transmembrane domain and a tetratricopeptide (TPR) domain, followed by its FIC domain [45]. Unlike FIC domains found in pathogenic bacteria, FICD is regulated to modulate cellular processes rather than to promote toxicity. Structural and sequence analysis of FIC domains revealed an inhibitory helix containing (S/T)xxxE(G/N) motif, where the glutamate residue forms a salt bridge with an arginine in the FIC motif [46]. When the glutamate is mutated, disrupting the salt bridge, the arginine co-ordinates the γ-phosphate of ATP, positioning the α-phosphate for the nucleophilic attack by the hydroxyl group of the substrate. In agreement with these structural studies, the E234G mutation in FICD resulted in significantly enhanced auto-AMPylation activity [46]. This discovery opened avenues for future studies to investigate FIC-mediated AMPylation in human cells without the constraints of autoinhibition.

FICD localizes to the lumen of the endoplasmic reticulum (ER) where it AMPylates heat shock protein 70 cognate 3 (also known as immunoglobulin binding protein, BiP), a key regulator of the unfolded protein response (UPR) [47,48]. As a molecular chaperone, BiP plays a critical role in protein quality control by facilitating the translocation and folding of newly synthesized proteins [3]. Post-translational modification of BiP was reported as early as 1983 by multiple laboratories (reviewed in [3]); however, its precise nature remained unknown for decades. In 2014–2015, seminal studies from the Orth, Mattoo, and Ron laboratories identified FICD-mediated AMPylation as a key regulatory mechanism of BiP function [47–49].

Under conditions of low ER stress, BiP is AMPylated by FICD to inhibit its chaperone activity. However, during periods of heightened stress when misfolded proteins accumulate, AMPylation is reversed, leading to BiP activation to restore substrate binding and folding [3]. The reversibility of BiP AMPylation underscores how post-translational modifications orchestrate adaptive responses to cellular stress. Interestingly, biochemical and cellular studies demonstrated that FICD is a bifunctional enzyme which acts as a deAMPylase or AMPylase [50,51]. Similar to the accessory domains found in bacterial FIC proteins, the TPR domain of FICD mediates substrate recognition by facilitating the binding of BiP and positioning it for FIC motif-mediated catalysis [45,52,53]. Furthermore, glutamate 234 plays a catalytic role in deAMPylation by acting as a general base to deprotonate a water molecule for the nucleophilic attack on the phosphodiester bond, thereby hydrolyzing BiP-AMP and restoring the functional state of BiP [52].

The deAMPylation reaction does not simply reverse AMPylation. DeAMPylation results in the release of AMP, rather than the regeneration of ATP, underscoring the mechanistic divergence between these two enzymatic processes [50,54]. This indicates that the active site of FICD is capable of catalyzing two distinct reactions: adenylyl/AMP transfer (AMPylation) and phosphodiester bond hydrolysis (deAMPylation). While the regulatory mechanisms driving this functional switch *in vivo* remain unclear, studies suggest that factors such as the oligomeric state of FICD or the identity of active site metal ions may modulate its dual activity [51,55,56]. These concepts are reviewed in detail in [2,3]. Further investigation into these regulatory cues will uncover how AMPylation and deAMPylation fine-tune the function of BiP in response to ER stress.

## Selenoprotein O

Protein kinases catalyze the transfer of the γ-phosphate from ATP to substrates. The human genome encodes more than 500 protein kinases that regulate various essential cellular processes [57]. Approximately 10% of these kinases are predicted to be pseudokinases because they lack residues necessary for catalytic activity [58]. The majority of pseudokinases function as allosteric regulators, molecular switches, and signaling scaffolds or possess alternative transferase activities [59,60]. One such pseudokinase is selenoprotein O (SelO, SelenoO), which lacks the catalytic aspartate involved in phosphoryl transfer [61]. SelO is one of 25 proteins in humans that contain the 21^st^ amino acid selenocysteine [62]. Until recently, the enzymatic activity of SelO remained unknown despite being one of the most highly conserved members of the selenoprotein and protein kinase families [63].

The crystal structure of *Pseudomonas syringae* SelO revealed a core kinase fold with amino acid variations in its active site that facilitate an unconventional mode of ATP binding [5]. In contrast with canonical kinases which possess a hydrophobic pocket that stabilizes the adenine moiety of ATP, SelO co-ordinates ATP in an inverted orientation. This inversion is facilitated by a distinct arrangement of charged residues and metal ions in the active site, which primarily interact with the β- and γ-phosphate. Furthermore, the catalytic aspartate is shifted from its conserved position in the catalytic motif of kinases to a position proximal to the α-phosphate of ATP. These studies uncovered the structural basis for the enzymatic activity of SelO, which catalyzes the AMPylation of serine, threonine, and tyrosine residues of protein substrates, a previously unrecognized activity for a member of the protein kinase superfamily.

Unlike GS-ATase and FICD, which act on a single substrate, SelO can AMPylate multiple proteins involved in metabolism and the oxidative stress response [5,8]. One of the substrates of *E. coli* SelO is glutaredoxin 1 (grxA), a thioredoxin-like protein that catalyzes protein deglutathionylation by reducing glutathione-protein mixed disulfides [64]. SelO AMPylates tyrosine 13 in grxA, which is located near the glutathione-binding cysteine that is essential for deglutathionylation activity [5]. AMPylation of the conserved active site residue inhibits grxA activity, maintaining proteins in a glutathionylated state to prevent oxidative damage [5,65]. SelO-deficient *E. coli* and *Saccharomyces cerevisiae* display reduced protein S-glutathionylation levels and have decreased viability upon oxidative insult [5]. The *Salmonella typhimurium* homolog of SelO was shown to catalyze manganese-dependent UMPylation of bacterial chaperones such as DnaK and GroEl, reminiscent of the nucleotide diversity observed in FIC domains [66].

The human homolog of SelO contains an N-terminal mitochondrial targeting peptide, followed by a kinase-like domain and a highly conserved selenocysteine at its C-terminus [67]. Recent studies have uncovered the substrates of mammalian SelO and its functional importance in melanoma metastasis [8]. In a murine immunocompetent model of melanoma, SelO deficiency did not affect the growth of primary tumors but substantially reduced the frequency of melanoma cells in the blood and the total metastatic burden in visceral organs. Such impaired metastasis is partially rescued by antioxidant treatment, suggesting that SelO deficiency impairs metastasis at least partly by increasing oxidative stress. *In vitro* AMPylation assays using mitochondria isolated from tumors revealed that SelO catalyzes the AMPylation of mitochondrial aconitase and succinate dehydrogenase subunit A, which are key components of the TCA cycle and the electron transport chain. In concordance with this, melanoma cells lacking SelO display increased activity of mitochondrial complex II [8]. Although these results uncover a critical role for SelO in the mitochondria, the AMPylation activity of SelO remains to be conclusively linked to physiological substrate modification in cells.

## SidM

*Legionella pneumophila*, the bacterium responsible for Legionnaire’s disease, is an intracellular pathogen that establishes a replication vacuole within host cells [68]. To support its survival and replication during infection, *L. pneumophila* secretes an array of bacterial effector proteins that manipulate host cell signaling pathways [69]. One such effector protein is SidM, also known as DrrA, which recruits Rab1 GTPase to the *Legionella*-containing vacuole (LCV) [70,71]. SidM is a multidomain protein containing an N-terminal ATase domain with the G-X_11_-D-X-D catalytic motif that shares structural similarity with GS-ATase [72,73]. Following the ATase domain, SidM contains a guanine nucleotide exchange factor (GEF) domain and a phosphatidylinositol-4-phosphate (PI4P) binding domain, which, together, enable its interaction with Rab1 and localization to the LCV membrane [70,71,74].

The ATase domain of SidM AMPylates Rab1 at tyrosine 77 in the switch II region to lock Rab1 in its GTP-bound state, preventing its removal by GTPase-activating proteins (GAPs) and GDP dissociation inhibitors (GDIs) [72,75]. While SidM promotes the retention of Rab1 on the LCV in the initial stages of infection, *L. pneumophila* later removes Rab1 from the LCV by translocating LepB, a GAP effector [76]. The antagonistic functions of SidM (GEF) and LepB (GAP) depict a tightly controlled mechanism by which *L. pneumophila* dynamically remodels the LCV during the infection. Since AMPylation blocks Rab1 inactivation by GAPs, Rab1 must be deAMPylated before it can be targeted by LepB. This necessity suggests that *L. pneumophila* has a dedicated deAMPylase for regulating Rab1 cycling during infection.

In bacterial genomes, functionally related genes often cluster together within operons. Notably, SidD, a putative effector protein, is encoded adjacent to SidM, hinting at a potential functional relationship [77]. The conservation of SidD and SidM across multiple *Legionella* species further supports the notion that they act in a co-ordinated manner [77]. In support of this hypothesis, the deletion of SidD from *L. pneumophila* abolished Rab1 de-AMPylation activity [77,78]. Furthermore, co-expression of SidD rescued the growth defect associated with SidM expression, reinforcing SidD’s role in counteracting SidM-mediated AMPylation [78]. Mechanistically, SidD catalyzes the hydrolytic cleavage of Rab1-AMP to release unmodified Rab1 and AMP [77].

Structural analysis revealed that SidD contains a binuclear metal center, with two Mg^2+^ ions co-ordinated by conserved aspartate residues in the active site [79]. While SidD displays similarity to metal-dependent protein phosphatases, it also contains unique sequence insertions and structural rearrangements around the catalytic site [79]. These structural adaptations are hypothesized to regulate substrate binding and catalysis, suggesting that SidD has evolved a specialized function as a deAMPylase. These findings highlight the molecular mechanisms that *L. pneumophila* employs to regulate host Rab1 in a reversible manner to establish a replicative niche [80].

## Significance of AMPylation in health and disease

Protein AMPylation is emerging as a prominent post-translational modification used by bacterial effectors to alter host cell signaling, as exemplified by SidM and various FIC domain-containing effectors [81]. Beyond bacterial pathogens, the eukaryotic AMPylases FICD and SelO have been implicated in essential cellular stress responses, including the UPR and oxidative stress regulation [2].

While the loss of FICD is well tolerated under normal growth conditions, studies in multiple cell lines, *Drosophila melanogaster*, *Caenorhabditis elegans*, and mice demonstrate the importance of FICD under conditions of ER stress [47,82–88]. Recently, a cohort of unrelated patients with motor neuron disease was identified to harbor an R374H mutation in FICD [7]. Arginine 374 co-ordinates ATP in the active site and forms the inhibitory salt bridge with glutamate 234 [45,46,89]. Fibroblasts from patients with the R374H mutation exhibit increased levels of AMPylated BiP, suggesting that the mutation decreases the deAMPylation activity [7]. Another mutation in the active site, R371S, has been linked to infancy onset diabetes mellitus and neurodevelopmental delay [6]. R371S exhibits weak AMPylation activity while inhibiting deAMPylation, leading to an accumulation of AMPylated BiP, as observed with the R374H mutation [6]. Expression of FICD R371S in CHO cells reduced the secretion of the reporter protein, secreted embryonic alkaline phosphatase (SEAP). These observations suggest a potential mechanism underlying the diabetic phenotype observed in patients with the R371S mutation [6]. While the complete loss of FIC is tolerated, mutations that result in the accumulation of AMPylated, inactive BiP are linked to pathological conditions. Collectively, these pathologies highlight the importance of BiP regulation in proteostasis and human physiology.

## Conclusion and future perspectives

Although AMPylation was discovered more than 50 years ago, a significant portion of the research was conducted in the past couple of decades and much remains to be learned about this emerging PTM. Interestingly, a recent report demonstrates that FICD can catalyze AMPylation using non-canonical nucleotides such as diadenosine tetraphosphate, raising the possibility of alternative substrates to ATP [90]. Furthermore, studies on SelO and FICD suggest that the activity of AMPylases may be regulated, making AMPylation more difficult to detect under normal conditions [5,50,86]. Thus, novel strategies are needed to enrich and identify AMPylated proteins in cells without the need for exogenous labeling.

Several methods have been developed to study AMPylation, including radiolabeled [α-^32^P] ATP, N^6^ labeled ATP analogs, mass spectrometry, and monoclonal AMPylation antibodies [91–96]. While biotinylated and fluorescently tagged ATP have been used to detect AMPylation, their bulky tags may interfere with the activity of some AMPylases [5,50]. To overcome this limitation, click chemistry has been widely used to derivatize the adenine moiety and label AMPylated substrates [91,95–97]. These methods are reviewed in [54].

**Figure 3:**
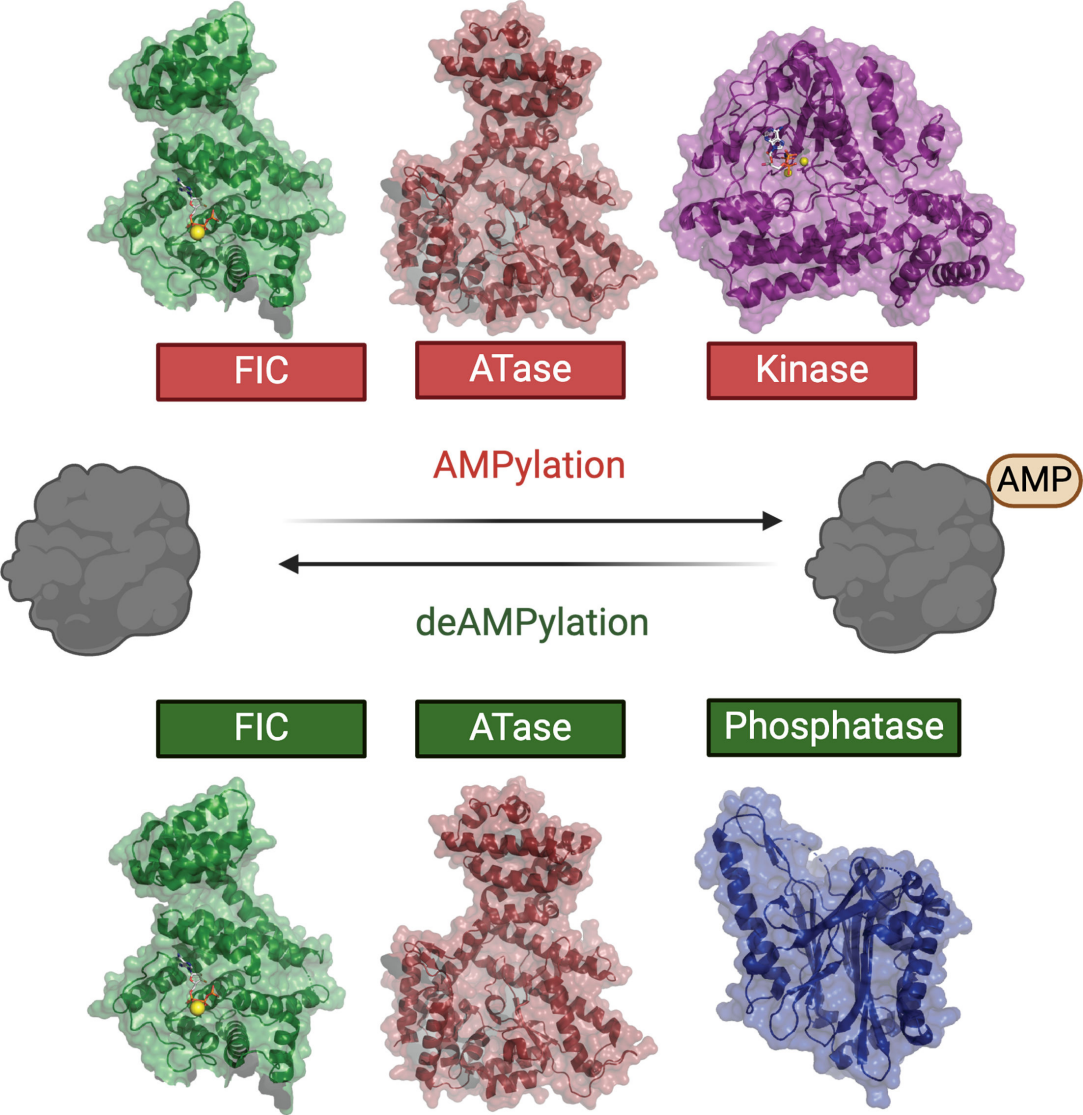
Structural diversity in AMPylases. Examples of the three folds that catalyze AMPylation: Adenylyltransferase (GS-ATase), FIC (FICD), and kinase domains (SelO). The three folds that catalyze deAMPylation are the bifunctional FIC and ATase domains, as well as the phosphatase fold (SidD). Surface representations of *P. syringae* SelO (PDB ID: 6EAC), *Homo sapiens* FICD (PDB ID: 4U07), and *E. coli* GS-ATase C terminal (PDB ID: 3K7D), and *L. pneumophila* SidD (PDB ID: 4IIP). Metals are shown as yellow spheres. Image generated with BioRender. AMP, adenosine monophosphate; FIC, filamentation induced by cyclic AMP; ATase, adenylyltransferase.

The identification of AMPylases has resulted from studies of nitrogen metabolism in *E. coli*, pathogenic bacterial effector proteins, and pseudokinases [5,11,12,38,72]. The discovery of AMPylation is somewhat serendipitous, emerging from investigations across diverse biological contexts rather than a directed search for this post-translational modification. As our understanding of AMPylation grows, it is evident that this modification may be more widespread than previously recognized, exhibiting remarkable structural diversity (Figure 3) [44]. The nucleotidyltransferase fold found in GS-ATase and SidM shares homology with DNA polymerase β [73]. In contrast, the FIC proteins exhibit no sequence similarity to adenylyltransferases and catalyze a diverse range of modifications, including the covalent addition of AMP, UMP, and phosphocholine [98–100]. Similarly, the deAMPylation reaction is also catalyzed by structurally diverse folds. SidM harbors a phosphatase fold, while FICD and GS-ATase catalyze both AMPylation and deAMPylation [27,50,79]. Although FICD is a bifunctional enzyme like GS-ATase, key differences exist. FICD catalyzes hydrolytic deAMPylation, while GS-ATase mediates a phosphorolytic reaction [50]. Moreover, FICD uses the same active site, while GS-ATase uses separate active sites linked by a regulatory domain [27,50]. These examples highlight the structural diversity in AMPylation.

In conclusion, AMPylation is a reversible and dynamic modification that provides valuable insight into the intricate regulatory mechanisms governing cellular function. Further research into the mechanisms and substrates of AMPylation will undoubtedly shed light on the complexity of cellular signaling and host–pathogen interactions.

**Perspectives**

- Protein AMPylation is an evolutionarily conserved post-translational modification that regulates protein function and signaling in both prokaryotic and eukaryotic systems.
- The reversibility of AMPylation suggests that it functions to fine-tune the activity of eukaryotic cellular signaling. Although widely studied in the context of bacterial pathogenesis, recent biochemical and proteomic studies suggest that many eukaryotic AMPylated substrates remain unidentified.
- Improvements in the current methods for studying AMPylation may reveal novel AMPylases and deAMPylases, shedding light on their roles in health and disease. A deeper understanding of the physiological importance of AMPylation across various tissues and disease states may drive translational applications.

## Abbreviations

AMP: adenosine monophosphate
ER: endoplasmic reticulum
FIC: filamentation induced by cyclic AMP
GAPs: GTPase-activating proteins
GEF: guanine nucleotide exchange factor
GS: glutamine synthetase
GS-ATase: glutamine synthetase adenylyltransferase
LCV: *Legionella*-containing vacuole
SelO: selenoprotein O
TPR: tetratricopeptide
UPR: unfolded protein response
grxA: glutaredoxin 1
